# Lean mass is the strongest predictor of bone mineral content in type-2 diabetes and normal individuals: an eastern India perspective

**DOI:** 10.1186/s40200-014-0090-5

**Published:** 2014-09-02

**Authors:** Indira Maisnam, Deep Dutta, Satinath Mukhopadhyay, Subhankar Chowdhury

**Affiliations:** Department of Endocrinology & Metabolism, Institute of Post-Graduate Medical Education and Research (IPGMER) and Seth Sukhlal Karnani Memorial (SSKM) Hospital, 244 AJC Bose Road, Calcutta, 700020 India

**Keywords:** Bone mineral content,bone mineral density, Type-2 diabetes, Lean mass, Fat mass

## Abstract

**Background:**

Impact of body fat distribution on bone mineral content (BMC) and density (BMD) at different sites has not been studied in type-2 diabetes (T2DM). This study aimed to compare BMC and BMD in normal (BMI < 25 kg/m^2^) and increased BMI (BMI ≥ 25 kg/m^2^) T2DM patients with age and BMI matched normal controls, and evaluate the impact of lean mass and body fat distribution parameters on them.

**Methods:**

Seventy-six T2DM patients and 56 normal controls underwent anthropometric assessment, blood sampling and estimation of BMC, BMD, body fat and lean mass distribution by dual energy X-ray absorptiometry (DXA).

**Results:**

Increased BMI individuals (n = 63) had significantly higher BMD, BMC, fat mass and significantly lower 25-hydroxy-vitamin-D (25OHD), as compared to normal BMI individuals (n = 69). Lean mass had stronger positive correlation with BMC and BMD, compared to fat mass. BMI, sagittal abdominal diameter (SAD) and Android/Gynoid (A/G) ratio had positive correlation with BMC and BMD. Percent body fat had negative correlation with BMC and BMD. T2DM patients had higher central obesity (A/G ratio). WC was the best predictor of A/G ratio. Regression analysis revealed lean mass to be the strongest predictor of BMC after adjusting for age, sex, BMI and 25OHD, in normal individuals and patients with diabetes, followed by fat mass. BMD right femur, BMC, lean mass and A/G ratios were significantly higher in males (n = 74). Fat mass and percent body fat were significantly higher in females (n = 58). Fat mass was the best predictor of BMC in males where as lean mass was the best predictor of BMC in females.

**Conclusion:**

Increased BMI and T2DM are associated with increased BMC and BMD at different sites, with lean mass having the strongest impact on BMC in normal individuals and patients with diabetes. Males have higher BMC and BMD as compared to females, likely due to a greater lean mass, A/G ratio, along with lesser fat mass and percent body fat.

## Introduction

Type-2 diabetes (T2DM) has been associated with increased fracture risk with relative risk varying from 1.2-2.2 in different studies [1–3]. Besides the increased risk of falls in T2DM patients secondary to hypoglycemia, neuropathy and retinopathy, altered bone strength is believed to play a role in this increased fracture risk. Bone mineral density (BMD), though not the best, is the most commonly used indirect measure of bone strength and fracture risk. However studies assessing BMD in T2DM have given mixed results with few suggesting normal to increased BMD in T2DM [4,5] where as others documenting decreased BMD [6]. Factors modulating BMD in T2DM have not been well determined. Obesity has a complex effect on BMD. Increased mechanical loading of bones along with increased anabolic effects of increased interaction of insulin with IGF-1 receptor on osteoblasts is believed to increase BMD [7]. In contrast, increased sequestration of vitamin-D in obesity worsens vitamin-D deficiency impairing bone health [8]. Further, increased visceral adipose tissue is believed to have a negative effect on BMD [9]. Data on impact of body fat and lean mass distribution on BMD is available predominantly from post-menopausal women with or without osteoporosis [10–15]. Such studies among patients with diabetes are scanty, and are complicated by lack of adjustment for duration of diabetes, type of diabetes, use of glitazones and insulin.

Hence this study aimed to compare the total body bone mineral content (BMC) and BMD in recently diagnosed T2DM patients (<6 months diagnosis of diabetes) with BMI matched normal controls, and to evaluate the impact of body fat and lean mass on BMC. We also planned to evaluate the role of anthropometric measurements [waist circumference (WC), and sagittal abdominal diameter (SAD)] in predicting central adiposity as determined by android/gynoid (A/G) ratio.

## Methods

Stable patients of recently diagnosed T2DM (≤6 months of diagnosis), on oral anti-diabetes medications and family members of patients of T2DM, 35–55 years age, attending the diabetic clinic were considered for the study. The study duration was from August 2010 till July 2013. Patients of type-1 diabetes mellitus, secondary diabetes mellitus, monogenic diabetes, post-menopausal women, comorbid states like chronic kidney disease, liver disease, autoimmune and rheumatologic disorders, malignancies, history of insulin use, pioglitazone, drugs which interfere with BMD (oral contraceptives for more than 6 months in last 2 years, glucocorticoids, immunosuppressive agents, bisphosphonates, lithium), alcohol abuse, cigarette smoking in the last 6 months, obesity syndromes, severe obesity (BMI > 35 kg/m^2^), pregnant women, perimenopausal or postmenopausal women were excluded. Family members of T2DM patients who had persistently normal fasting blood glucose (FBG) and 2-hour post glucose blood glucose (2hPGBG) on 75-gram glucose OGTT done twice over a period of one week were considered as normal controls. The study protocol was explained to all the considered individuals and only those who gave informed written consent were included. All subjects enrolled in this study were from the same geographical area (Gangetic Delta, Eastern India). The institutional ethics committee approved the study. Approval from the institutional ethics committee of IPGMER & SSKM hospital was obtained before initiation of the study.

The included individuals attended the outpatient services of the department after an overnight fast (12 hours). They under-went detailed clinical evaluation, anthropometric assessment blood samples collected, serum separated and stored at −80° for further analysis. Height was measured to the nearest 0.1 cm using a Charder HM200PW wall-mounted stadiometer [calibrated using a 36″ calibration rod (Perspective Enterprise, Portage, Michigan, USA)], and body weight was measured in light clothing to the nearest 0.1 kg using an electronic calibrated scale (Tantia, Japan, Model-HA521, Lot number-860525). Waist circumference was measured without clothes in the measurement area to the nearest 0.1 cm by a measuring tape at the level of the mid-point of the costal margin and the highest point of the iliac crest after a normal expiration. SAD was measured to the nearest 0.1 cm in the supine position with bent knees on a firm examination table after a normal expiration and without clothes in the measurement area. At the level of iliac crest (L_4–5_) SAD was measured using a sliding-beam caliper as the distance between the examination table up to the horizontal level, where the caliper arm touched the abdomen slightly but without compression. Several studies have suggested SAD to be a strong predictor of visceral adiposity [16–18].

Serum 25-hydroxy-vitamin-D (25OHD) was estimated using chemiluminescent microparticle immunoassay (Architect 25-OH Vitamin D assay, Abbott, USA). The analytical sensitivity of the assay was 4 ng/ml with a range of 9.4 ng/ml to 165.5 ng/ml. The CV of the assay ranged from 2.8-4.6%. Patients were considered vitamin D deficient if serum levels of 25OHD were <20 ng/ml, insufficient if levels 20-30 ng/ml and sufficient if levels >30 ng/ml [19]. FBG, 2hPGBG, lipid profile, liver function, calcium, phosphorus, and creatinine were estimated using clinical chemical analyzer (Daytona, serial number-58260536, Furuno Electric, Nishnomeya, Japan). DXA was done with a GE Lunar DPX NT densitometer by the same technician to determine BMD (g/cm^2^) at the lumbar spine (L_1_-L_4_) and both neck of femur, BMC (g) total body fat (g), percentage fat mass, lean body mass (g), android fat (g) and gynoid fat (g). The lower border of android region was set at upper border of pelvis. The upper border of android region was set at a level being 20% of distance from upper border of pelvis to neck. The upper border of gynoid region was set at level below the pelvis at which the distance from the upper border of the pelvis to the upper border of gynoid region was 1.5 times the length of the android region. The lower border of the gynoid region was set at level at which the length of gynoid region was twice the length of android region [20]. Quality control procedures were done as per manufacturer’s recommendations. Coefficient of variation for DXA ranged from 0.7 to 3.1%.

The T2DM (Group-A) and normal individuals (Group-B) were divided into 2 groups, normal BMI (Group-A1 and Group-B1) and increased BMI (overweight to obese) (Group-A2 and Group-B2) based on BMI of <25 and ≥25 respectively, for analysis [20].

### Sample size calculation

Data on BMD in T2DM is conflicting ranging from decreased, normal to increased BMD [4–6]. In contrast, type-1 diabetes (T1DM) is uniformly associated with decreased BMD. Using DXA, osteopenia has been reported in 60% of patients with T1DM [21]. Using this data, it was calculated that at least 65 T2DM patients needs to be evaluated, to keep the power of the study at 80%, type-1 error at 5% and precision either side of the proportion at 10%.

### Statistical analysis

Student’s *t* test was used for analysis of continuous variables. Fisher’s exact test was used for binary variable. χ2 test was used for categorical variables. One-way ANOVA with post-hoc analysis was used to study outcomes where 3 or more groups were present. All values are expressed as mean ± standard deviation. P < 0.05 has been considered statistically significant. Stepwise multiple linear regression models were used to estimate the regression coefficients for parameters showing significant bivariate association with BMC and BMD after adjusting for age, BMI and vitamin-D.

## Results

Seventy-six recently diagnosed T2DM patients and 56 normal controls who fulfilled all criteria were included in this study, which consisted of 74 males and 58 females. Clinical and anthropometric parameters like age (44.21 ± 6.13 vs. 44.75 ± 3.9 years; P = 0.610), BMI (24.61 ± 3.30 vs. 24.70 ± 3.60 kg/m^2^; P = 0.890), waist circumference (89.56 ± 9.90 vs. 89.73 ± 10.92 cm; P = 0.933) and SAD (20.32 ± 2.27 vs. 20.28 ± 2.38 cm; P = 0.932) were comparable among patients with diabetes as compared to controls. Systolic blood pressure (131.26 ± 18.4 vs. 117.95 ± 11.57 mm of Hg; P < 0.001) but not diastolic blood pressure (78.00 ± 9.46 vs. 75.63 ± 5.96 mm of Hg; P = 0.138) was significantly higher in patients with diabetes as compared to controls. Mean HbA1c in T2DM patients was 7.56 ± 1.27% as compared to 5.22 ± 0.14% in controls.

Of the patients with T2DM 31 had increased BMI and 45 had normal BMI. Among normal controls, 32 had increased BMI, and 24had normal BMI. Serum 25OHD was significantly lower in increased BMI individuals as compared to normal BMI individuals, both among diabetes patients and controls (Table 1). Increased BMI individuals had significantly higher BMD at bilateral neck of femur and lumbar spine as compared to their normal BMI counterparts, both in T2DM patients and controls (Table 1). In increased BMI individuals, BMC was significantly higher compared to their normal BMI counterparts, in T2DM but not controls (Table 1). Fat mass and percent body fat were also significantly higher in increased BMI individuals as compared to their normal BMI counterparts (Table 1). Lean mass was not significantly different among increased BMI individuals as compared to normal BMI individuals (Table 1).

**Table 1:** **Comparison of anthropometric, biochemical, bone mineral and body fat distribution parameters in normal BMI (<25 kg/m** ^**2**^ **) vs. increased BMI (≥25 kg/m** ^**2**^ **) individuals (type-2 diabetes and normal controls respectively)**

**Parameter**	**Normal BMI T**_**2**_ **DM (n = 45) Group-A1**	**Increased BMI T**_**2**_ **DM (n = 31) Group-A2**	**Normal BMI Controls (n = 24) Group-B1**	**Increased BMI Controls (n = 32) Group-B2**	**P value**
Age (years)	44.14 ± 5.89	44.32 ± 6.58	46.75 ± 2.02^†^	42.35 ± 4.31^†^	0.060
Sex (Male: Female)	22:24	15:15	18:6	19:13	0.147
BMI (kg/m^2^)	22.3 ± 1.67^‡^	27.8 ± 2.10^‡^	21.9 ± 1.50^†^	28.2 ± 2.65^†^	<0.001
Waist circumference (cm)	84.5 ± 7.87^‡^*	96.8 ± 7.86^‡^	79.3 ± 3.50^†^*	98.2 ± 6.04^†^	<0.001
SAD (cm)	19 ± 1.79^‡^*	21.9 ± 1.87^‡^	18 ± 0.34^†^*	22.1 ± 1.40^†^	<0.001
Systolic BP (mmHg)	129 ± 19*	133 ± 16	110 ± 2.9^†^*	128 ± 11.12^†^	<0.001
Diastolic BP(mmHg)	77 ± 9	79 ± 9	73 ± 4.30^†^	79 ± 6.73^†^	0.032
FBG (mg/dl)	142 ± 56*	133 ± 41^#^	81 ± 14.17*	84 ± 7.26^#^	<0.001
2 hr-PGBG (mg/dl)	207 ± 78*	208 ± 70^#^	111 ± 10.75*	115 ± 13.8^#^	<0.001
HbA1c (%)	7.5 ± 1.2*	7.6 ± 1.3^#^	5.2 ± 0.11*	5.2 ± 0.17^#^	<0.001
25OHD (ng/ml)	23.7 ± 8.0	17.0 ± 12.8^#^	24.7 ± 7.49^†^	11.1 ± 4.18^†#^	<0.001
Calcium (mg/dl)	9.2 ± 0.47	9.2 ± 0.51	9.4 ± 0.63^†^	8.9 ± 0.43^†^	0.009
Phosphorus (mg/dl)	3.8 ± 0.43^‡^	4.0 ± 0.43^‡^	4.07 ± 0.42	4.1. ± 0.28	<0.001
ALP (U/l)	159 ± 82*	157 ± 55	106 ± 17.49*	120 ± 40.31	0.001
Total cholesterol (mg/dl)	175 ± 41	163 ± 43	165 ± 16.10	120 ± 40.85	0.395
Triglyceride (mg/dl)	145 ± 63	167 ± 89	114 ± 37.61	142 ± 46.90	0.036
LDL-C (mg/dl)	105 ± 28	122 ± 31^#^	104 ± 9.38	115 ± 34.6^#^	0.039
HDL-C (mg/dl)	44 ± 6*	46 ± 11	40 ± 4.09^†^*	37 ± 6.11^†^	<0.001
Creatinine (mg/dl)	0.86 ± 0.16	0.86 ± 0.12	0.9 ± 0.12	0.8 ± 0.08	0.286
ALT (U/l)	29 ± 14	31 ± 11	29 ± 0.25	39 ± 17.55	0.165
BMD (RF) (g/cm^2^)	0.95 ± 0.125^‡^	1.11 ± 0.15^‡^	0.9 ± 0.11^†^	1.07 ± 0.17^†^	<0.001
BMD (LF) (g/cm^2^)	0.97 ± 0.11^‡^*	1.13 ± 0.13^‡^	0.88 ± 0.11^†^*	1.08 ± 0.18^†^	<0.001
BMD (LS) (g/cm^2^)	1.05 ± 0.12^‡^	1.22 ± 0.16^‡^	1.01 ± 0.05^†^	1.14 ± 0.15^†^	<0.001
% Body Fat	32 ± 7.29^‡^	40 ± 6.09^‡^	34.08 ± 3.55^†^	45.96 ± 6.42^†^	<0.001
Fat mass (kg)	16.29 ± 3.95^‡^	27.06 ± 7.49^‡^	18.17 ± 1.95^†^	30.73 ± 6.85^†^	<0.001
Lean mass (kg)	34.72 ± 7.09	36.92 ± 7.48	35.82 ± 7.66	36.20 ± 7.31	0.206
BMC (kg)	2.17 ± 0.32^‡^	2.5 ± 0.40^‡^	2.28 ± 0.36^†^	2.58 ± 0.62^†^	<0.001
Android/Gynoid	1.17 ± 0.15*	1.19 ± 0.25	1.05 ± 0.09*	1.11 ± 0.1	0.022

T_2_DM: Type 2 Diabetes Mellitus; BMI: Body Mass Index; SAD: Sagittal Abdominal Diameter; BP: blood pressure; FBG: Fasting blood glucose; 2 h-PGBG: 2-hour post-glucose blood glucose; HbA1c: glycosylated hemoglobin; 25OHD: 25-hydroxy-vitamin-D; LDL: Low Density Lipoprotein Cholesterol; HDL: High Density Lipoprotein Cholesterol; BMD: Bone Mineral Density; BMC: Bone Mineral Content; ALT: alanine aminotransferase; LS: lumbar spine; LF: left femur; RF: right femur; all values expressed as mean ± standard deviation; P-value calculated by one way ANOVA with least significant difference post-hoc analysis; P < 0.05 considered statistically significant; ^‡^: P < 0.05 when comparing normal BMI with increased BMI diabetes patients; ^†^: P < 0.05 when comparing normal BMI with increased BMI controls; *: P < 0.05 when comparing normal BMI diabetes patients with normal BMI controls; ^#^: P < 0.05 when comparing increased BMI diabetes patients with increased BMI controls.

Normal BMI diabetes patients had higher waist circumference and SAD, as compared to normal BMI controls, which approached statistical significance (Table 1). BMD left femur was significantly higher in normal BMI T2DM patients as compared to controls (Table 1). Normal BMI T2DM patients had higher Android to Gynoid fat (A/G) ratio, which approached statistical significance (Table 1). BMD lumbar spine was significantly higher in increased BMI T2DM patients as compared to increased BMI normal controls (Table 1). Fat mass and percent body fat were significantly higher in increased BMI controls as compared to increased BMI diabetes patients (Table 1). Normal BMI diabetes patients had decreased lean mass as compared to normal BMI controls, but not statistically significant.

A significantly greater proportion of females had diabetes as compared to males (67.24% vs. 50%) (Table 2). Males had significantly higher waist circumference and SAD in spite of comparable BMI (Table 2). Serum 25OHD was significantly lower, serum LDL-C and HDL-C were significantly higher in females. BMD right femur, BMC, lean mass and A/G ratios were significantly higher in males, where as fat mass and percent body fat were significantly higher in females (Table 2).

**Table 2:** **Comparison of anthropometric, biochemical, bone mineral and body fat distribution parameters in males vs. females**

**Parameter**	**Males (n = 74)**	**Females (n = 58)**	**P-value**
Diabetes: Normal controls	37:37	39:19	0.047
Age (years)	46.15 ± 1.09	42.32 ± 5.16	0.001
BMI (kg/m^2^)	24.45 ± 3.35	24.82 ± 3.51	0.57
Waist circumference (cm)	92.56 ± 9.7	86.22 ± 9.15	0.001
SAD (cm)	20.88 ± 2.29	19.67 ± 2.19	0.005
Systolic BP (mmHg)	123.91 ± 16.04	129.08 ± 18.88	0.111
Diastolic BP(mmHg)	75.61 ± 7.04	78.65 ± 9.48	0.048
FBG (mg/dl)	109.48 ± 42.95	126.10 ± 54.05	0.071
2 hr-PGBG (mg/dl)	156.03 ± 65.45	193.92 ± 79.48	0.008
HbA1c (%)	6.39 ± 1.52	6.92 ± 1.46	0.067
25OHD (ng/ml)	24.01 ± 11.02	20.06 ± 8.46	0.043
Calcium (mg/dl)	9.31 ± 0.60	9.09 ± 0.40	0.068
Phosphorus (mg/dl)	3.68 ± 0.54	3.59 ± 0.46	0.411
ALP (U/l)	130.60 ± 70.94	139.12 ± 42.64	0.529
Total cholesterol (mg/dl)	164.65 ± 29.78	178.77 ± 44.01	0.062
Triglyceride (mg/dl)	150.63 ± 60.81	138.58 ± 60.77	0.358
LDL-C (mg/dl)	99.48 ± 22.60	111.90 ± 32.23	0.029
HDL-C (mg/dl)	39.48 ± 8.79	45.49 ± 7.68	0.001
Creatinine (mg/dl)	0.93 ± 0.16	0.81 ± 0.12	0.001
ALT (U/l)	33.11 ± 13.11	31.12 ± 15.42	0.581
BMD (RF) (g/cm^2^)	1.04 ± 0.19	0.97 ± 0.12	0.022
BMD (LF) (g/cm^2^)	1.05 ± 0.2	0.99 ± 0.12	0.087
BMD (LS) (g/cm^2^)	1.12 ± 0.16	1.10 ± 0.17	0.441
% Body Fat	32.53 ± 6.36	41.21 ± 6.19	0.001
Fat mass (kg)	20.49 ± 6.58	23.39 ± 8.78	0.052
Lean mass (kg)	41.43 ± 5.54	30.44 ± 4.17	0.001
BMC (kg)	2.65 ± 0.50	2.08 ± 0.27	0.001
Android/Gynoid	1.25 ± 0.17	1.04 ± 0.13	0.001

BMI: Body Mass Index; SAD: Sagittal Abdominal Diameter; BP: blood pressure; FBG: Fasting blood glucose; 2 h-PGBG: 2-hour post-glucose blood glucose; HbA1c: glycosylated hemoglobin; 25OHD: 25-hydroxy-vitamin-D; LDL: Low Density Lipoprotein Cholesterol; HDL: High Density Lipoprotein Cholesterol; BMD: Bone Mineral Density; BMC: Bone Mineral Content; ALT: alanine aminotransferase; LS: lumbar spine; LF: left femur; RF: right femur; all values expressed as mean ± standard deviation; P-value calculated by unpaired t-test; P < 0.05 considered statistically significant.

Lean mass had a stronger positive correlation with whole body BMC and BMD at different sites as compared to fat mass, after adjusting for age, BMI and 25OHD, both in normal individuals and patients with diabetes (Table 3). BMI, SAD and A/G ratio also had a positive correlation with BMC and BMD at different sites (Table 3). Percent body fat had negative correlation with BMC and BMD at different sites. 25OHD had positive correlation with BMC and BMD at different sites only in normal controls (Table 3). Waist circumference most strongly correlated with A/G ratio (r = 0.56; P < 0.001 and r = 0.55; P < 0.001), followed by SAD (r = 0.49; P < 0.001 and r = 0.42; P = 0.007), after adjusting for BMI, in patients with diabetes and controls respectively.

**Table 3:** **Correlation between bone mineral density at different sites and total bone mineral content (BMC) with anthropometric parameters, and body fat and lean mass distribution parameters in diabetes patients and normal controls, after adjusting for age, body mass index (BMI) and 25-hydroxyvitamin-D**

**Parameter**	**BMD (RF)**	**BMD (LF)**	**BMD (LS)**	**BMC**
T2DM patients(n = 74)				
Age	0.11	0.10	0.1	0.3*
BMI	0.4*	0.32 ^#^	0.29*	0.34^#^
25OHD	0.1	0.1	0.1	0.18
HbA1c	0.08	0.13	0.13	−0.03
SAD	0.27	0.27	−0.16	0.38^#^
Fat Mass	0.18	0.17	0.1	0.24
Lean Mass	0.38*	0.35*	0.05	0.42^#^
% body fat	−0.31*	−0.32*	−0.12	−0.34^#^
A/G Ratio	0.28*	0.27*	−0.01	0.39^#^
Normal Controls (n = 58)				
Age	−0.16	−0.25	−0.32	0.14
BMI	0.35	0.26	0.11	0.30*
25OHD	0.33^#^	0.35^#^	0.35^#^	0.21^#^
HbA1c	0.18	0.16	0.16	0.10
SAD	0.27	0.36*	0.41*	0.37*
Fat Mass	0.12	0.05	0.14	0.21
Lean Mass	0.35*	0.33*	0.44^#^	0.31*
% body fat	−0.29*	−0.24*	−0.25*	−0.33^#^
A/G Ratio	0.31^#^	0.28*	0.21*	0.33^#^

T2DM: Type-2 diabetes; BMD: bone mineral density; RF: right femur; LF: left femur; LS: lumbo-sacral spine; BMC: total body bone mineral content; SAD: Sagittal abdominal diameter; A/G: Android/Gynoid fat ratio; Pearson’s correlation coefficient calculated; value in parentheses represent P-value; P < 0.05 considered statistically significant; *: P < 0.05; ^#^: P < 0.001.

For parameters showing significant bivariate association with BMC and BMD at different sites (BMI, SAD, lean mass, fat mass, percent body fat and A/G ratio), generalized linear models were constructed to determine their potential independent contributions to BMC and BMD after adjusting for age, BMI, 25OHD and HbA1c in Model 1, and age, sex, BMI and 25OHD in Model 2. Multiple linear regression analysis revealed that lean mass was the strongest predictor of whole body BMC followed by fat mass, both in normal individuals and patients with diabetes (Table 4). Percent body fat, SAD and A/G ratio were predictive of BMC only in normal individuals. Fat mass was the best predictor of BMC in males where as lean mass was the best predictor of BMC in females (Table 4). Similar predictions were not observed for BMD at different sites.

**Table 4:** **Multiple linear regressions showing the effect of body fat distribution parameters on bone mineral content (BMC) after adjustment for variables in different models**

	**Male (n = 74)**		**Female (n = 58)**		**Normal Individuals (n = 56)**		**Type-2 diabetes (n = 76)**	
	**Model-1**		**Model-1**		**Model-2**		**Model-2**	
	**β**	**P-value**		**P-value**	**β**	**P-value**	**Β**	**P-value**
Total body BMC								
SAD	−0.048	0.565	0.141	0.422	0.185	0.01	0.028	0.727
Lean mass	0.130	0.150	0.224	0.008	0.177	0.01	0.207	0.001
Fat mass	0.112	0.032	0.054	0.422	0.106	0.033	0.151	0.012
Tissue fat percent	0.085	0.511	0.060	0.634	0.171	0.02	0.091	0.336
A/G ratio	0.073	0.223	−0.019	0.635	0.176	0.051	−0.116	0.158

Stepwise regression analysis was done adjusting for the following variables: Model-1: adjusted for age, BMI, vitamin-D and HbA1c; Model-2: adjusted for age, sex, BMI and vitamin-D; BMC: bone mineral content; SAD: sagittal abdominal diameter; A/G ratio: android/gynoid ratio.

## Discussion

Lower vitamin-D in increased BMI individuals in our study can be explained by the increased sequestration of vitamin-D in the adipose tissue [8]. Adipocytes are believed to have some role in vitamin-D destruction and it has been suggested that vitamin-D deficiency per se leads to worsening of obesity by directly stimulating lipogenesis and by inhibiting catecholamine induced lipolysis [22,23]. Polymorphisms in vitamin D gene and its receptor have been linked to obesity [24–26]. The lower vitamin-D levels in increased BMI controls in our study as compared to increased BMI diabetes patients can be explained by the higher body fat content in increased BMI controls.

Our study showed that patients with diabetes (normal and increased BMI) had higher BMC and BMD as compared to normal individuals. Similarly individuals with increased BMI (with or without diabetes), in spite of a lower vitamin-D state, had higher BMC and BMD as compared to normal BMI individuals. This is similar to observations from Rotterdam study that noted that BMD at both lumbar spine and femoral neck was substantially higher in patients with diabetes as compared to normal individuals, even after adjusting for confounding variables [4]. The Rancho Bernardo study also documented increased BMD in women with diabetes as compared to normal individuals [27]. Increased anabolic effect of hyperinsulinemia in T2DM may have some role in the increased BMC and BMD in T2DM [7]. Increased mechanical loading of the bones secondary to obesity may explain the increased BMC and BMD in obesity. Also increased aromatase activity of adipose tissue may also contribute to this increased BMC and BMD in obesity [28]. De Laet et al., in a meta-analysis of 12 multinational cohorts comprising nearly 60,000 adult subjects, showed that independent of sex, those with BMI > 25 kg/m^2^ had significantly higher BMD and lower rates of hip, osteoporotic, and all fractures [28].

Waist circumference and SAD (measures of central obesity) were almost significantly higher in normal BMI T2DM and compared to BMI matched controls. Our study showed that WC correlated most strongly with A/G ratio followed by SAD. Hence WC may be a better predictor of central adiposity as compared to SAD. Increased A/G ratio, the measure of central adiposity was almost significantly higher in normal BMI diabetes patients as compared to controls. Similarly decreased total body fat along with comparable WC and SAD in increased BMI diabetes patients as compared to increased BMI controls is suggestive of a higher percentage of visceral adiposity in increased BMI diabetes patients. Increased A/G ratio in increased BMI diabetes supports this hypothesis. Hence diabetes (normal or increased BMI) is associated with increased central adiposity. Truncal fat mass has been shown to correlate strongly with insulin resistance in mildly overweight new onset diabetes [29]. In our study A/G ratio had a positive correlation with BMC and BMD at different sites both in diabetes patients and normal controls. This is in accordance with previous observations of subjects with android fat distribution having higher BMD as compared to those with gynoid distribution [30]. It is believed that increased insulin resistance associated with increased central obesity leads to decreased sex-hormone binding globulin, leading to increased free testosterone and estradiol promoting bone formation [30]. Also it is believed that increased android fat leads to increased mechanical loading of femur leading to increased BMD [30]. It must be clarified that central adiposity is constituted by visceral adipose tissue (VAT) and subcutaneous adipose tissue (SAT). Increased SAT is believed to have a positive effect on BMD, where as increased VAT is believed to have a negative effect on BMD [9]. Increased VAT is associated with increased circulating inflammatory cytokines and chemokines, altered profile of leptin and adiponectin leading to increased osteoclast activation and bone loss [9]. However VAT and SAT were not separately evaluated in our study and is one of the limitations of this study. Use of DXA is limited by its ability to measure only the total body fat and it cannot differentiate between VAT and SAT. Another limitation of this study is its cross-sectional nature and lack of measurement of cytokines and adipokines.

Our study showed that increase in fat mass is associated with increased BMC both in diabetes and normal individuals. This is in accordance with recent reports from India, Holland and Korea showing increased fat mass having a positive effect on BMD at all sites in normal men and women [31].The most important observation of this study is the strongest correlation of lean mass with BMC and BMD both in normal individuals and patients with diabetes, with regression analysis confirming lean mass to be the strongest predictor of BMC, after adjusting for age, sex, BMI and vitamin-D. Increased lean mass perhaps results in more mechanical loading of body as compared to fat mass, resulting in a greater increase in bone mass [32,33]. It has been suggested that bone adapts more to dynamic muscle load than to static load, explaining the stronger effect of lean mass over fat mass on BMC [34]. This explains the beneficial effect of physical activity on bone health. Isolated increase in fat mass (adiposity) results in lesser dynamic loading of bones secondary to decreased physical activity. Additional benefits of increased lean mass include prevention of falls, thus decreasing fracture risk. Increased occurrence of diabetes among females in our study cohort explains the higher blood glucose parameters and HbA1c in females. Central obesity was more common in males, who also had higher BMC and lower fat mass and body fat percent, supporting the previous hypothesis (vide supra).

To the best of our knowledge, this is the first study to report BMC, BMD and body composition changes in normal and increased BMI T2DM patients comparing it with BMI matched controls. Our study showed that both T2DM and increase in BMI are associated with increased BMC and BMD, with lean mass having the strongest impact on BMC. Further prospective studies are suggested to document the beneficial effects of increased physical activity on increased lean mass, and its impact on long term fall and fracture outcomes in diabetes.

## Conclusions

Both increased BMI and T2DM are associated with increased BMC and BMD at different sites, with lean mass having the strongest impact on BMC in normal individuals and patients with diabetes followed by fat mass. Males have higher BMC and BMD as compared to females, likely due to greater lean mass, A/G ratio, along with lesser fat mass and percent body fat. Fat mass was the best predictor of BMC in males where as lean mass was the best predictor of BMC in females.

## References

[1] Bonds DE, Larson JC, Schwartz AV, Strotmeyer ES, Robbins J, Rodriguez BL, Johnson KC, Margolis KL (2006). Risk of fracture in women with type 2 diabetes mellitus: the women’s health initiative observational study. J Clin Endocrinol Metab.

[2] Janghorbani M, Feskanich D, Willett WC, Hu F (2006). Prospective study of diabetes and risk of hip fracture: the nurses’ health study. Diabetes Care.

[3] Strotmeyer ES, Cauley JA, Schwartz AV, Nevitt MC, Resnick HE, Bauer DC, Tylavsky FA, de Rekeneire N, Harris TB, Newman AB (2005). Non-traumatic fracture risk in type 2 diabetes mellitus and impaired fasting glucose in older white and black adults: the health, aging and body composition study. Arch Intern Med.

[4] De Liefde II, der KliftM V, De Laet CE, van Daele PL, Hofman A, Pols HA (2005). Bone mineral density and fracture risk in type-2 diabetes mellitus: the Rotterdam study. Osteoporos Int.

[5] Ma L, Oei L, Jiang L, Estrada K, Chen H, Wang Z, Yu Q, Zillikens MC, Gao X, Rivadeneira F (2012). Association between bone mineral density and type 2 diabetes mellitus: a meta-analysis of observational studies. Eur J Epidemiol.

[6] Dutta MK, Pakhetra R, Garg MK (2012). Evaluation of BMD in type 2 diabetes mellitus before and after treatment. Med J Armed Forces India.

[7] Zhao N, Tang XL, Zhen DH, Liu HH (2013). Higher calcaneal bone mineral density in men with metabolic syndrome in a Chinese population. J Diabetes.

[8] Blum M, Dolnikowski G, Seyoum E, Harris SS, Booth SL, Peterson J, Saltzman E, Dawson-Hughes B (2008). Vitamin D3 in fat tissue. Endocrine.

[9] Russell M, Mendes N, Miller KK, Rosen CJ, Lee H, Klibanski A, Misra M (2010). Visceral fat is a negative predictor of bone density measures in obese adolescent girls. J Clin Endocrinol Metab.

[10] Taaffe DR, Villa ML, Holloway L, Marcus R (2000). Bone mineral density in older non-Hispanic Caucasian and Mexican-American women: relationship to lean and fat mass. Ann Hum Biol.

[11] Hsu YH, Venners SA, Terwedow HA, Feng Y, Niu T, Li Z, Laird N, Brain JD, Cummings SR, Bouxsein ML, Rosen CJ, Xu X (2006). Relation of body composition, fat mass, and serum lipids to osteoporotic fractures and bone mineral density in Chinese men and women. Am J Clin Nutr.

[12] El Hage R, Jacob C, Moussa E, Baddoura R (2011). Relative importance of lean mass and fat mass on bone mineral density in a group of Lebanese postmenopausal women. J Clin Densitom.

[13] Dytfeld J, Ignaszak-Szczepaniak M, Gowin E, Michalak M, Horst-Sikorska W (2011). Influence of lean and fat mass on bone mineral density (BMD) in postmenopausal women with osteoporosis. Arch Gerontol Geriatr.

[14] Liu S, Li J, Sheng Z, Liao E (2011). Relationship between body composition and age, menopause and its effects on bone mineral density at segmental regions in Central Southern Chinese postmenopausal elderly women with and without osteoporosis. Arch Gerontol Geriatr.

[15] Yoo HJ, Park MS, Yang SJ, Kim TN, Lim KI, Kang HJ, Song W, Baik SH, Choi DS, Choi KM (2012). The differential relationship between fat mass and bone mineral density by gender and menopausal status. J Bone Miner Metab.

[16] Kvist H, Chowdhury B, Grangård U, Tylén U, Sjöström L (1988). Total and visceral adipose tissue volumes derived from measurements with computed tomography in adult men and women: predictive equations. Am J Clin Nutr.

[17] Risérus U, Arnlöv J, Brismar K, Zethelius B, Berglund L, Vessby B (2004). Sagittal abdominal diameter is a strong anthropometric marker of insulin resistance hyperproinsulinemia in obese men. Diabetes Care.

[18] Guzzaloni G, Minocci A, Marzullo P, Liuzzi A (2009). Sagittal abdominal diameter is more predictive of cardiovascular risk than abdominal fat compartments in severe obesity. Int J Obes.

[19] Adams JS, Hewison M (2010). Update in vitamin D. J Clin Endocrinol Metab.

[20] Klein S, Fabrini E, Romijn JA, Melmed S, Polonsky KS, Larsen PR, Kronenberg HM (2011). Obesity. Williams textbook of endocrinology.

[21] Kemink SA, Hermus AR, Swinkels LM, Lutterman JA, Smals AG (2000). Osteopenia in insulin-dependent diabetes mellitus; prevalence and aspects of pathophysiology. J Endocrinol Invest.

[22] Le Roith D (2002). Beta-cell dysfunction and insulin resistance in type 2 diabetes: role of metabolic and genetic abnormalities. Am J Med.

[23] Mc Carty MF, Thomas CA (2003). PTH excess may promote weight gain by impending catecholamine-induced lipolysis-implications for the impact of calcium, vitamin D and alcohol on body weight. Med Hypotheses.

[24] Iyengar S, Hamman RF, Marshall JA, Majumder PP, Ferrell RE (1989). On the role of Vitamin D binding globulin in glucose homeostasis: results from the San Luis Valley diabetes study. Genet Epidemiol.

[25] Baier LJ, Dobberfuhl AM, Pratley RE, Hanson RL, Bogardus C (1998). Variations in the vitamin D-binding protein (Gc locus) are associated with oral glucose tolerance in non-diabetic Pima Indians. J Clin Endocrinol Metab.

[26] Hitman GA, Mannan N, McDermott MF, Aganna E, Ogunkolade BW, Hales CN, Boucher BJ (1998). Vitamin D receptor gene polymorphisms influence insulin secretion in Bangladeshi Asians. Diabetes.

[27] Barrett-Connor E, Holbrook TL (1992). Sex differences in osteoporosis in older adults with non-insulin-dependent diabetes mellitus. JAMA.

[28] De Laet C, Kanis JA, Oden A, Johanson H, Johnell O, Delmas P, Eisman JA, Kroger H, Fujiwara S, Garnero P, McCloskey EV, Mellstrom D, Melton LJ, Meunier PJ, Pols HA, Reeve J, Silman A, Tenenhouse A (2005). Body mass index as a predictor of fracture risk: a meta-analysis. Osteoporos Int.

[29] Bavenholm PN, Kuhl J, Pigon J, Saha AK, Ruderman NB (2003). EfedicS. Insulin resistance in type 2 diabetes: association with truncal obesity, impaired fitness, and atypical malonyl coenzyme A regulation. J Clin Endocrinol Metab.

[30] Heiss CJ, Sanborn CF, Nichols DL, Bonnick SL, Alford BB (1995). Associations of body fat distribution, circulating sex hormones, and bone density in postmenopausal women. J Clin Endocrinol Metab.

[31] Marwaha RK, Garg MK, Tandon N, Mehan N, Sastry A, Bhadra K (2013). Relationship of body fat and its distribution with bone mineral density in Indian population. J Clin Densitom.

[32] Zillikens MC, Uitterlinden AG, van Leeuwen JP, Berends AL, Henneman P, van Dijk KW, Oostra BA, van Duijn CM, Pols HA, Rivadeneira F (2010). The role of body mass index, insulin, and adiponectin in the relation between fat distribution and bone mineral density. Calcif Tissue Int.

[33] Kim CJ, Oh KW, Rhee EJ, Kim KH, Jo SK, Jung CH, Won JC, Park CY, Lee WY, Park SW, Kim SW (2009). Relationship between body composition and bone mineral density (BMD) in perimenopausal Korean women. Clin Endocrinol (Oxf).

[34] Lanyon LE, Rubin CT (1984). Static vs. dynamic loads has an influence on bone remodeling. J Biomech.

